# Effects of Video Game Training on Behavioral and Electrophysiological Measures of Attention and Memory: Protocol for a Randomized Controlled Trial

**DOI:** 10.2196/resprot.6570

**Published:** 2017-01-24

**Authors:** Soledad Ballesteros, Julia Mayas, Eloisa Ruiz-Marquez, Antonio Prieto, Pilar Toril, Laura Ponce de Leon, Maria L de Ceballos, José Manuel Reales Avilés

**Affiliations:** ^1^ Studies on Aging and Neurodegenerative Diseases Research Group Department of Basic Psychology II Universidad Nacional de Educación a Distancia Madrid Spain; ^2^ Facultad de Derecho Department of Social Work Universidad Nacional de Educación a Distancia Madrid Spain; ^3^ Cajal Institute Neurodegeneration Group, Departament of Translational Neurobiology and Biomedicine Research Center for Neurodegenerative Diseases Consejo Superior de Investigaciones Científicas Madrid Spain; ^4^ Facultad de Psicología Departamento Methodology of the Behavioral Sciences Universidad Nacional de Educación a Distancia Madrid Spain

**Keywords:** attention, C-reactive protein, cognitive training, healthy aging, inflammation, electrophysiology, video games, working memory

## Abstract

**Background:**

Neuroplasticity-based approaches seem to offer promising ways of maintaining cognitive health in older adults and postponing the onset of cognitive decline symptoms. Although previous research suggests that training can produce transfer effects, this study was designed to overcome some limitations of previous studies by incorporating an active control group and the assessment of training expectations.

**Objective:**

The main objectives of this study are (1) to evaluate the effects of a randomized computer-based intervention consisting of training older adults with nonaction video games on brain and cognitive functions that decline with age, including attention and spatial working memory, using behavioral measures and electrophysiological recordings (event-related potentials [ERPs]) just after training and after a 6-month no-contact period; (2) to explore whether motivation, engagement, or expectations might account for possible training-related improvements; and (3) to examine whether inflammatory mechanisms assessed with noninvasive measurement of C-reactive protein in saliva impair cognitive training-induced effects. A better understanding of these mechanisms could elucidate pathways that could be targeted in the future by either behavioral or neuropsychological interventions.

**Methods:**

A single-blinded randomized controlled trial with an experimental group and an active control group, pretest, posttest, and 6-month follow-up repeated measures design is used in this study. A total of 75 cognitively healthy older adults were randomly distributed into experimental and active control groups. Participants in the experimental group received 16 1-hour training sessions with cognitive nonaction video games selected from Lumosity, a commercial brain training package. The active control group received the same number of training sessions with The Sims and SimCity, a simulation strategy game.

**Results:**

We have recruited participants, have conducted the training protocol and pretest assessments, and are currently conducting posttest evaluations. The study will conclude in the first semester of 2017. Data analysis will take place during 2017. The primary outcome is transfer of benefit from training to attention and working memory functions and the neural mechanisms underlying possible cognitive improvements.

**Conclusions:**

We expect that mental stimulation with video games will improve attention and memory both at the behavioral level and in ERP components promoting brain and mental health and extending independence among elderly people by avoiding the negative personal and economic consequences of long-term care.

**Trial Registration:**

Clinicaltrials.gov NCT02796508; https://clinicaltrials.gov/ct2/show/NCT02796508 (archived by WebCite at http://www.webcitation.org/6nFeKeFNB)

## Introduction

### Background

Neurocognitive frailty is the biggest threat to successful aging [1]. There is overwhelming evidence that the number of inflammatory mediators increases vastly in pathological aging [2] and in healthy aging as well [3]. This inflammation has a negative impact on cognition [4]. C-reactive protein (CRP), an acute-phase protein of hepatic origin that increases following interleukin-6 secretion by macrophages and T cells, has been identified as a hallmark of systemic inflammation, and it can be assessed by noninvasive methods in saliva [5]. A key challenge that faces the aging society is to find new approaches to understand the aging mind and brain that could enhance successful, optimal aging [6]. Another major challenge is to understand the factors that could reduce or delay the negative consequences of age-related cognitive decline [7]. The idea behind this randomized controlled trial (RCT) is that cognitive abilities could be strengthened by interventions that promote positive brain plasticity [8].

Normal aging is associated with gray and white matter shrinkage. The lateral prefrontal cortex, cerebellum, and medial temporal lobe system including hippocampus are affected. Minimal reduction occurs in the entorhinal and occipital cortices [1,9,10]. These brain changes are associated with declines in several important cognitive functions, including processing speed, executive functions, working memory, and episodic memory [11-14]. However, other crystallized abilities such as general knowledge, verbal abilities [15-17], and implicit memory [18,19] are mostly preserved. Older adults with mild cognitive impairment [20] and Alzheimer disease patients have shown preserved implicit memory despite huge deteriorations in episodic memory [21,22]. Although behavioral priming for repeated pictures is spared in older adults, reduced neural activation, which is a signature of implicit memory, is affected. The relationship between brain function and behavior found in young adults is altered in older adults, although these age-related changes do not affect behavioral facilitation. These findings have implications for the notion that automatic processes, previously considered preserved with age, are susceptible to the effects of aging at the neural level. The age-invariant behavioral facilitation with stimulus repetition is observed as a result of more sustained neural processing of visual stimuli in older adults as a form of compensatory neural activity [23].

Electrophysiological studies have also found significant age-related event-related potential (ERP) changes in brain activity associated with memory retrieval in spite of similar performance in young and older adults [24]. Elders compensate for their lower level of parieto-occipital functioning as shown by smaller P300 amplitude at posterior sites by recruiting frontal sites as a mode of brain adaptation [25]. In touch, despite similar behavioral performance in a recognition memory task involving familiar 3D objects, young and older adults recruited different neural resources to perform the task. Age-related differences were found in brain oscillations, as measured by event-related spectral perturbations (ERSPs), suggesting the recruitment of additional neural resources as shown by greater alpha and beta power reductions in older adults [26]. Despite similar behavior performance, normal aging was found to affect ERPs and oscillatory brain activity during an incidental speeded haptic symmetry detection priming task. However, the young adults showed more positive amplitude than the older adults in the alpha and beta bands from stimulus onset [27]. All these neural results suggest that the older brain adapts in order to maintain the same level of performance as the younger brain both in explicit and implicit memory tasks.

Neural plasticity exists at several levels of the neural substrate [28,10], although not to the same degree in older as in younger adults [15,29,30]. These findings suggest that the aging brain retains some neuroplasticity, which can be influenced by the individual’s behavior. The prolonged mismatch between functional organismic supplies and environmental demands produces cognitive plasticity and underlines the capacity of the brain to implement behavioral flexibility [31]. Although there are personal factors, human behavior can change with appropriate training as the brain reorganizes in response to the environment [32]. Based on the idea of neuroplasticity, different types of interventions have been developed to ameliorate cognitive and functional declines by strengthening social networking using new information and communication technology (ICT) [33-35] and cognitive skills training [36,37] and promoting an active lifestyle [38,39] and physical activity training [40,41].

As people age, they experience declines in attentional control, mediated by the dorsolateral prefrontal cortex, and in long-term memory functions, mediated by the medial temporal lobe and the hippocampus. These areas suffer the highest degree of age-related atrophy [9]. Moreover, the prefrontal cortex facilitates the organization and contextualization of incoming information and interacts with the hippocampus during working memory implementation [42,43]. This relationship is strengthened with age [44]. These findings are especially relevant due to the existing links between these basic cognitive abilities and everyday functioning. The malfunction of these basic abilities is a significant predictor of older adult difficulties with the instrumental activities of daily living, leading to loss of independence [45,46]. Therefore, it is of vital importance to pursue the question of whether cognitive decline can be reversed or delayed through cognitive training interventions [47].

Video games and other computerized programs are receiving increasing interest from cognitive and neuroscientists to investigate the possibility of transfer to untrained tasks [48-50]. Researchers are increasingly using new technology, including cognitive training platforms and video games, to investigate their impact on cognition [48-52]. Computer-based interventions have the advantage that they can be easily used by elderly people living either at home or in nursing homes and could be a good alternative to traditional training programs [53]. This RCT aims to assess the effectiveness of training older adults with digital nonaction video games.

Two challenges must be overcome in order to develop an effective cognitive aging intervention [54-55]: (1) transferring training gains to untrained tasks and (2) designing interventions that encourage compliance. Older adults prefer games that involve mental challenge [56-57], while studies with young adults have shown that fast-paced action games result in broader transfer effects. A “first-person shooter” video game is not an appropriate choice for older adults [58]. Nonaction video games have potential advantages as they are enjoyable, low-cost, and can be self-administered. Recent results from a systematic review [53] and meta-analyses [59-61] suggest that training elders with video games improves information processing, with unexpected larger effects in old-older adults than in young-older adults. Moreover, the results of a recent meta-analysis of action video game training showed that healthy adults benefit from training in overall and specific cognitive domains, but young adults benefit more than older adults [62]. These findings underscore the potential of video game training as an effective intervention tool for cognitive improvement. Results of studies on executive function and working memory are less consistent. It is possible that nonaction video games are not an effective means of improving or maintaining these functions in older adults. Games provide an enjoyable way of passing the time and of giving meaning to the day [56]. In short, video games can offer important benefits to older adults, bearing in mind that intervention compliance is a key factor in longitudinal training studies [54].

A previous RCT conducted in our laboratory investigated the effects of nonaction video game training on a series of cognitive functions that decline with age and subjective well-being. Two groups of older adults participated in the study: an experimental group that received the training and a noncontact control group. Groups were similar at baseline on demographics, vocabulary, global cognition, and depression status. The results showed improvements in the video game–trained group and no change in the passive control group in processing speed, attention, and immediate and delayed visual recognition memory and a trend to improve in 2 dimensions of the Well-being Scale (affection and assertivity) [63]. However, visuospatial working memory and executive control (shifting strategy) functions did not improve [49,64,65].

A recent longitudinal intervention study conducted to investigate the effects of video game training in healthy older adults showed that 15 1-hour training sessions with 6 nonaction video games produced significant improvements on 2 visuospatial working memory tasks and on episodic and short-term memory tasks. Some of these gains were maintained over a 3-month follow-up period [52]. In both previous studies, experimental groups were compared with passive control groups. To better attribute training-related improvement to the intervention and to avoid placebo effects [66], the current RCT compares performance on a series of attentional and visuospatial working memory tasks of an experimental group trained with selected nonaction video games from the commercial Lumosity computerized training program (www.lumosity.com) with that of an active control group carrying out the same number of training sessions with The Sims, a simulation strategy game in which the player takes control of the life of a character in everyday activities, and SimCity, a life simulation game in which the player is the mayor of a city that he or she must develop. Both groups used a mobile tablet device during the training sessions. These video games were chosen to train the active control group because this group should also play with engaging and challenging video games the same training hours, potentially equating the social contact experienced during training by experimental and active-control groups.

Training expectation is an important issue in cognitive training studies [51,67]. To address this issue, participants report their expectations (increase or decrease) regarding their task performance for any assessment task on a 5-point Likert-type scale. After training sessions 1, 8, and 16 (final), participants respond to training feedback questions about engagement and motivation for each training game. The objective was to find out whether both groups were similarly engaged and motivated during training.

In sum, the growth of ICT-based tools, along with their progressive implementation in all social groups, offers a great opportunity to develop low-cost preventive intervention programs able to reach the entire population. This can contribute to improving the quality of life of elderly people, with a substantial reduction in health care costs related to the loss of autonomy and independence in this population group [68]. In this RCT we investigate not only possible cognitive improvement in healthy older adults in selected cognitive domains that decline with aging but also possible neural changes of training using electrophysiological methods at pre- and posttraining and 6-month follow-up. The study also explores neuroinflammatory mechanisms underlying possible cognitive training-induced effects using noninvasive saliva-testing methods.

### Objectives and Hypotheses

The study has 3 main complementary objectives:

1. To examine the behavioral and electrophysiological effects of training older adults with nonaction video games on a series of cognitive tasks designed to assess attentional functions (mainly response inhibition, distraction, and alertness) and to maintain and update verbal and visuospatial working memory. We are also interested in investigating the sustainability of the transfer effects after a 6-month no-contact period.

2. To explore whether motivation, engagement, or expectations account for possible training-related improvements.

3. To examine whether inflammatory mechanisms assessed with noninvasive measurement of CRP in saliva impair cognitive training-induced effects. Inflammatory parameters in the participants obtained by noninvasive saliva-testing methods will be analyzed in biochemical assays to study their possible correlations with cognitive abilities.

We hypothesize that participants in the Lumosity group will improve selective attention assessed with oddball and Stroop tasks and working memory assessed with Corsi blocks and *n*-back tasks in comparison with participants in the active control group. From an electrophysiological perspective, we also hypothetize that in the oddball task the cognitive video game–trained group will improve both the ability to return attention to the relevant task after having been distracted by the novel stimulus (reorientation negativity [RON]) and the ability of the brain to pay attention to the novel stimulus (P300).

Intervention studies conducted with older adults have focused mostly on training a given cognitive function and then measuring the improvement in that function [69,29] and less often on possible changes in neural activity after training [70,71]. Based on the idea that the older human brain still has some plasticity (the ability to change in response to external stimulation), our main hypothesis is that training older adults with appropriate, adapted, and motivating nonaction video games will transfer to cognitive functions that decline with age, such as attention and working memory, by improving these pillars of cognition, which are crucial to maintaining independent living and cognitive health in old age.

## Methods

### Overview of the Study Design

**Figure 1:**
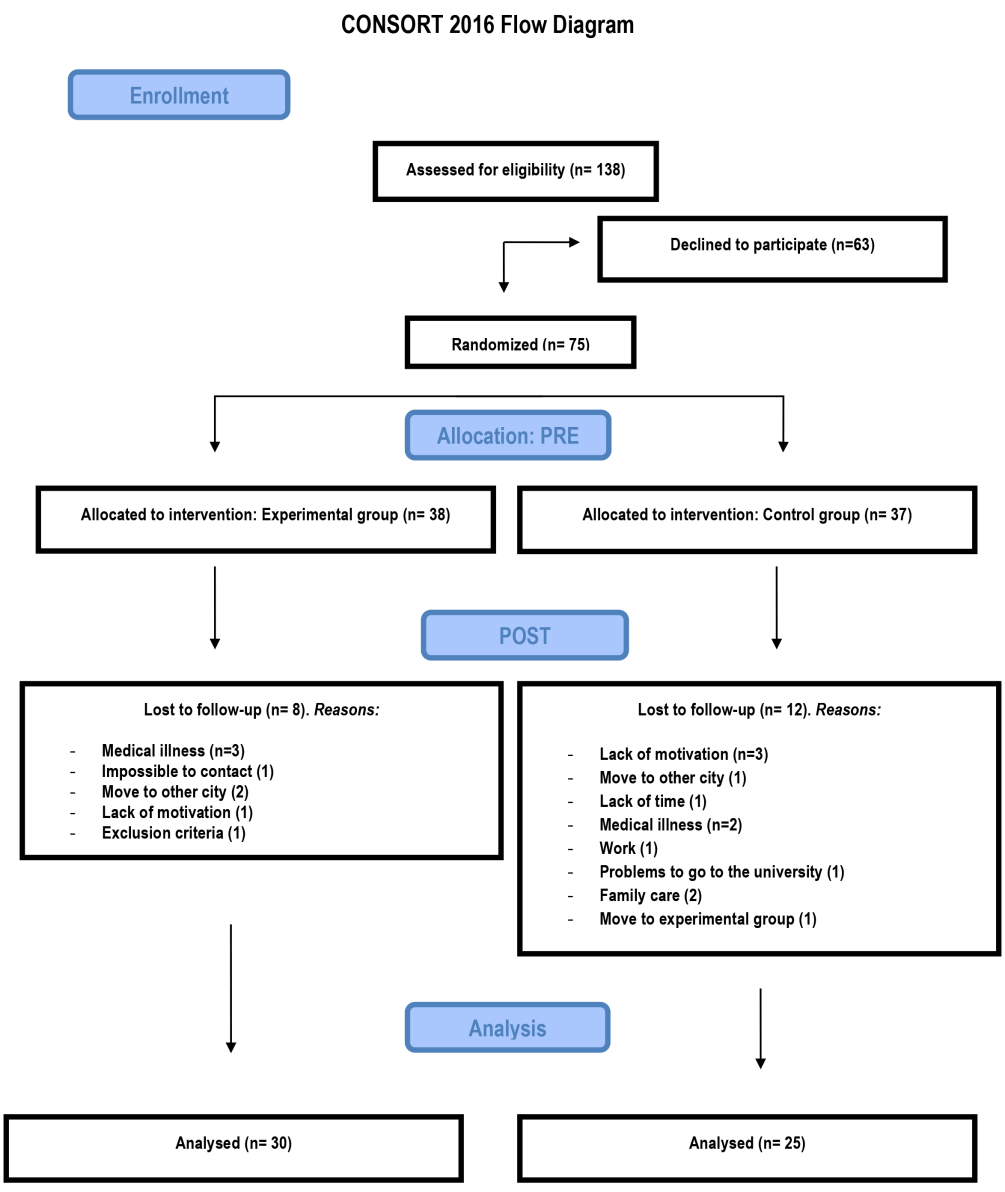
The Consolidated Standards of Reporting Trials flow diagram.

A 2-arm, parallel-design RCT [72] was designed to evaluate the effectiveness of video game training to promote cognitive and brain health in older adults. Figure 1 shows the Consolidated Standards of Reporting Trials flow diagram of the study. This RCT is being conducted in Madrid, Spain, with an allocation ratio of 1:1 to evaluate the influence of training with nonaction video games on attention and memory functions. The design was a single-blind design. Eligible participants were randomized using a computer algorithm into intervention (cognitive nonaction video game training) or active control (life-simulation games) groups. Researchers were not blind to treatment allocation. The Universidad Nacional de Educación a Distancia (UNED) Institutional Review Board approved the study protocol. All participants provided written informed consent.

### Eligibility and Exclusion Criteria

All participants live independently with normal or corrected-to-normal hearing and vision and are free of neurological or psychiatric disorders or traumatic brain injury. To determine their eligibility, each participant completed a screening battery consisting of the Mini-Mental State Examination (cut-off score 26) [73] to rule out possible cognitive impairment, the Yesavage Depression Scale (cut-off score less than 5) [74], and the Information subscale of the WAIS-III scale (normal score) [75]. Exclusion criteria were a diagnosis of dementia, cognitive impairment (score of <26 on the Mini-Mental State Examination), depression, less than 20/60 vision with or without correction, inability to complete the study activities, communication problems, or current plans to move to another city. All participants were healthy volunteers.

### Sample Size

The sample size is based on the number of observations needed to compare the 2 groups of gamers, those trained with Lumosity and those trained with The Sims. As we do not have standard deviations of the scores in our main dependent variables, we used G*Power 3.1 (Department of Psychology) to compute the number of participants, using analysis of variance as a statistical test. For this, using a power of .80, effect size of 0.50, an alpha level of .05, and 2 groups within the *F* test family, we obtained a required total sample size of 57. With a dropout rate of 10%, a total of 63 participants would be required. Rounding up the numbers, we would need more than 64 participants (32×2). As the flow diagram shows, the final number of participants matches these a priori computations closely: 38 participants were assigned to the experimental group (8 lost at posttest) and 37 to the control group (12 lost at posttest).

### Participants

Participants were randomly assigned to 1 of the 2 parallel groups, either the intervention video game training group or the active control group (see Figure 1). The pretest, posttest, and 6-month follow-up assessments are conducted at the UNED Department of Basic Psychology II by one of the authors with the assistence of other members of the group. The training sessions were carried out at the UNED Associate Center, Escuelas Pías, Madrid, and lasted 8 to 10 weeks. A total of 138 participants were recruited from flyers, advertisements, by word of mouth, and from UNED’s special program for the elderly screened for eligibility. Finally, 75 older adults (age range 55 to 84 years) met the requirements for participation in the study and signed the consent form and were randomly assigned to the experimental or the active control groups before carrying out the laboratory tasks. The 2 groups do not differ significantly in age, years of education, global cognition, depression, memory, or verbal abilities.

### Electroencephalograph Acquisition

While performing the experimental tasks, continuous electroencephalograph (EEG) activity is recorded using a NuAmps amplifier (Neuroscan Inc) located inside a soundproof, electrically shielded room. A 34-channel elasticized Quik-Cap with Ag/AgCl sintered electrodes (Neuroscan Inc) is used to record EEG data from scalp electrodes positioned according to the extended international 10-20 system (American Medical EEG Association, 1991). To control for the influence of ocular artifacts, vertical and horizontal electrooculograms are recorded in 2 bipolar channels. Eye blinks and vertical eye movements are monitored via electrodes located below and on the supraorbital ridge of the left eye. Horizontal artifacts are monitored via electrodes on the outer canthus of each eye. Linked mastoids (A1, A2) are used as reference, and participants are grounded to the AFz electrode. All data will be digitized using a NuAmps amplifier in continuous recording mode. Sampling rate will be 250 Hz, and all channels will be online band-pass filtered (0.1-70 Hz) and notch filtered (50 Hz) to eliminate power line artifacts. Analysis of the EEG recordings will be performed using the EEGLAB toolbox [76] and ERPLAB plugin for EEGLAB [77] both running under MATLAB environment (The MathWorks Inc). Continuous data will be filtered offline using a digital Butterworth filter (0.1-40 Hz; 12 dB per octave roll-off), an infinite impulse response filter that achieves a given filtering characteristic using less memory and fewer calculations than a similar finite impulse response filter. After filtering, data will be separated into baseline corrected and nonoverlapping epochs time-locked to the target onset (in both Stroop and oddball tasks) ranging from 200 ms before to 1000 ms after the onset of the target with the prestimulus interval (200 ms) as baseline period. Epochs containing high amplitude/frequency and muscle or other irregular artifacts will be removed by visual inspection. Only artifact-free epochs from correct trials will be selected for averaging. The existence of blinks and other ocular movements will not be a criterion for epoch rejection. This kind of artifact will be eliminated using Infomax Independent Component Analysis (ICA) decomposition [78-82]. We will use the default extended-mode *runica* training parameters [83], an extension of the original algorithm [78]. This extended mode allows a wider range of source signals (both super- and sub-Gaussian) while maintaining simplicity. Stopping weight change will be set to 1e–7. This rather conservative criterion for stopping learning lengthens ICA training, enabling cleaner and more reliable decompositions, particularly with more than 33 channels and limited number of epochs. After submitting epochs to ICA decomposition, artifactual components will be removed by inspection of their scalp topography and spectral power [84,27].

### General Procedure

Participants in the study were randomly assigned to the cognitive nonaction video game training group or the noncognitive game training active control group. The introduction of an active control group is necessary to avoid placebo effects [54]. Based on the results of our meta-analytic study [61], we use training regimes that are not too long to avoid loss of motivation, and simple, nonaction games that appeal more to elders. According to the temporal discount hypothesis [85], future rewards are less valuable than inmediate rewards. In each session, the trainees play 10 nonaction video games from Lumosity, described below.

The main question examined in our study is whether group (experimental group, active control group) interacts with testing session (pretest, posttest, and follow-up) with regard to performance on a series of cognitive tasks. At least 2 measures for each cognitive domain are acquired to examine transfer of training to untrained tasks demanding attention and working memory. We selected these cognitive domains because these functions deteriorate with aging and tax functions that are critical for independent living. To explore the underlying neural mechanisms of successful transfer of training gains to attentional mechanisms (alert, distraction, and inhibitory effects) and executive control (spatial working memory), electrophysiological data (ERPs) are recorded at pretraining (T1), posttraining (T2), and 6-month follow-up (T3).

All the methodological designs of the primary outcome measures have been constructed using the rules of counterbalancing and stimulus rotation. Response keys are counterbalanced across conditions. The computerized tasks have been programmed using E-Prime 2.0 (Psychology Software Tools Inc). All the statistical analyses of the behavioral results will be performed using SPSS (IBM Corp) and results will be considered significant at *P*<.05, with Bonferroni-corrected post hoc tests performed as appropriate. Continuous EEG activity are recorded in our laboratory with thin electrodes from 34 scalp sites using a NuAmps amplifier as mentioned before.

After randomization and initial assessment (baseline), participants were invited to attend the University Center of Escuelas Pias in Madrid to perform the training sessions in small groups. Trained and active control groups followed 16 training sessions carried out on different hours and days that lasted approximately 10 to 12 weeks.

Table 1 presents a short summary of the video games played by the experimental (trained) group. During the training sessions, participants in the experimental group play 10 nonaction video games selected from Lumosity.

The active control group carried out 16 training sessions with The Sims and SimCity BuildIt. Table 2 presents a short summary of these video games.

**Table 1:** Short description of the 10 video games played by the experimental group.

Game name	Trained function	Description
Tidal Treasures	Working memory	Player chooses objects and memorizes their choice.
Pinball Recall	Working memory	Player predicts a ball’s path.
Playing Koi	Divided attention	Player feeds fish and remembers which have already been fed.
Star Search	Selective attention	Player chooses the odd one out in a group of objects.
Lost in Migration	Selective attention	Player swipes in the direction the middle bird is facing in a flock of birds that appears on the screen.
Color Match	Response inhibition	Player compares one word's meaning to another word's color.
Disillusion	Task switching	Player matches tiles with different shapes, colors, or symbols.
Ebb and Flow	Task switching	Player swipes in the direction leaves are moving or pointing.
Highway Hazards	Information processing	Player races a car across the desert avoiding colliding with obstacles.
Speed Match	Information processing	Player determines whether a card appearing on the screen is the same as or different than the previous one.

**Table 2:** Life simulation games from Electronic Arts Inc played by the active control group.

Game name	Trained function	Description
SimCity BuildIt	None	Life simulation game in which the player is the mayor of a city that he or she must expand.
The Sims	None	Life simulation game in which the player creates characters (The Sims) that live in a virtual world that is similar to the real one. The Sims work, build their own homes, develop relationships, etc.

### Ethical Issues

This clinical trial is registered on the ClinicalTrials.gov database [NCT02796508] [72]. The UNED Ethical Review Board approved the trial. All the participants gave their written informed consent before the study started and were informed of their right to terminate participation at any time. The work described has not been published previously.

### Measurement of C-Reactive Protein in Saliva by Enzyme-Linked Immunosorbent Assay

Several parameters influencing inflammation (eg, infections, diabetes) are obtained from the participants: height and weight are recorded to obtain the body mass index (gross obesity could increase inflammation), drugs ingested (anti-inflammatories, antibiotics, antioxidants), possible infections and when they happened (last month, last week, or today), smoking habit (which may increase CRP levels in saliva), and defective oral health (eg, bleeding gums). Participants are asked not to eat or smoke at least 30 minutes before saliva collection. Following a mouth rinse with water, 1 mL of saliva is collected with a straw into a tube containing 2% ethylenediaminetetraacetic acid as preservative. Samples are stored at −80ºC until assayed. Assessment of CRP levels is performed with an enzyme-linked immunosorbent assay (ELISA) obtained from Salimetrics according to the manufacturer instructions. A saliva sample collected from participants at pretest, posttest, and follow-up assessments will be analyzed with ELISA in order to correlate the quantity of CRP with task performance.

### Primary Outcomes

#### Effects of Video Game Training on Attentional Networks: Behavioral and Electrophysiological Results

##### Oddball Task

The capacity to suppress irrelevant information and to concentrate on the relevant task [86] is negatively affected by aging. The question is whether the effects of training with cognitive video games are transferable to untrained tasks (transfer effect). Despite the great appeal of video games as a way to improve perceptual and cognitive abilities, evidence of their efficacy is mixed [49,50,52,64,87-88]. However, the results of several recent meta-analytic studies [59-62] suggest that video game training can be moderately effective in healthy older adults but that its positive effects are moderated by several variables including the complexity of the games, the age of the participants, the duration of the training program, and the cognitive processes assessed.

Previous studies indicate that deviance distraction occurs because deviant sounds violate the cognitive system’s expectations [89]. Attention capture has been studied from an electrophysiological perspective; the distractor is characterized by a pattern of 3 brain responses [90,91]: (1) mismatch negativity (MMN) and enhanced N1 when the distractor deviates considerably from the repetitive background, (2) P3a (novelty P3), and (3) RON. The MMN response reflects the preattentive detection of an unexpected change in the auditory context and results from the comparison between a memory trace for past acoustic stimuli and the current auditory signal [92]. The P3a response represents the involuntary orientation of attention toward the novel sound [93] and results from an attentional interruption involving frontal areas [94]. RON is also observed when participants are performing a primary task and must redirect their attention toward that task [95]. In this RCT, we undertake electrophysiological recordings to explore the underlying neural mechanisms of successful transfer of training to attentional performance in the oddball task. We expect that the cognitive video game–trained group will improve both the ability to return attention to the relevant task after having been distracted by the novel stimulus (RON) and the ability of the brain to pay attention to the novel stimulus (P300). In our lab, we previously investigated the effect of novelty in young adults using a haptic oddball paradigm consisting of processing textured surfaces [96]. We do not expect this pattern of results in the active control group and thus predict a group × session interaction.

##### Stroop and Negative Priming Task

Cognitive processes that involve top-down control mechanisms decline with aging but more automatic processes do not [20,22]. The Stroop interference effect reflects the extra time needed to resolve the conflict generated by the irrelevant word meaning in the incongruent condition. One view is that suppression processes relying on executive control are engaged in preventing the irrelevant dimension from taking control of the response [97]. In this study, we investigate the effect of training older adults with nonaction video games on inhibition processing using both behavioral and electrophysiological responses. The task has been designed to assess training-related behavioral and neural changes in Stroop interference and in negative priming (NP). NP (a measure of distractor inhibition) and the standard Stroop effect are assessed in the same task. In the standard NP procedure, participants are presented with pairs of prime and probe displays containing 2 stimuli, the to-be-responded target and the to-be-ignored distractor. In the critical trials, participants must respond to a target that served as a distractor in the previous prime display (the ignored repetition condition). The common finding is that reaction times to targets in the ignored repetition condition are slower than in the control condition, in which the distractor in the prime display is not repeated as the target in the probe display [98-101]. The aim is to investigate whether training older adults improves control, effortful inhibition (Stoop interference), to a greater extent than automatic passive inhibition (NP). The experimental task uses ERPs in combination with the Stroop and NP before and after training to examine the neural correlates of inhibition [102]. Responses for the Stroop analysis will be coded as a function of the congruency between the color and the meaning of the stimulus. Congruent trials are those in which the color of the word coincides with the color in which it is presented. Incongruent trials are those in which the color word does not coincide with the color in which it is displayed. We will also code trials according to the congruency of the previous trial (N−1) in order to compute the NP effect for each trial. The design for the NP effect consists of Group (younger, older adults), Session (pretraining, posttraining, follow-up), and Type of intervention (experimental, active control) as between-participants factors and Repetition (ignored repetition and control) as within-participants factors. Responses for NP analyses will be coded as a function of the relationship between the color of the current target word and the color denoted by the word in the previous trial (distractor). Thus, the ignored repetition trials are those in which the word in the preceding trial denoted the color of the word of the current stimulus. Control trials are those in which both the target (color) and the distractor (word) in the current trial are different from the target and distractor in the previous trial. The ignored repetition condition is always an incongruent trial preceded by an incongruent trial. The aim is to investigate whether the Stroop interference effect in older adults decreases after training while NP does not change. An important addition to the behavioral measures will be the ERP recording while performing the task before and after training to investigate brain activity changes after training.

#### Effects of Training on Spatial Working Memory

##### Overview

To investigate whether training transfers to improvements in spatial working memory, the participants perform a computerized Corsi blocks task. Recent meta-analytic studies conducted with trainees from childhood to older adults [60] and with older adults [61] found negligible effects of video game training on executive functions. The absence of improvement after training may depend on the specific kind of video games used. Studies that used first-person shooter action games reported benefits after training [103,104]. This type of game requires great perceptual abilities, preparedness for unpredictability, and strong emphasis on peripheral visual processing [105]. However, older adults do not like this type of game [55]. Previous findings from our lab yielded mixed results. One study [49] showed no transfer to working memory tasks, while a second study [52] showed that the trainees’ performance improved significantly after training in 2 visuospatial working memory tasks, the Corsi blocks and the jigsaw puzzle. Our results contrast with those of Nouchi et al [88] who found improvements in executive functions after training for 5 weeks. The ineffectiveness of training in spite of the fact that several of the nonaction video games mimic working memory tasks is in line with the findings of Boot and colleagues [54]. Due to these mixed results, the aim of this RCT is to find out whether training effects transfer to the spatial working memory of older adults.

##### Corsi Blocks

The original task [106] consists of a set of 9 identical blocks (3 x 3 x 3 cm) irregularly positioned on a wooden board (23 x 28 cm). The participant is required to point to the blocks in their presentation order. The length of the block sequences increases until recall is no longer correct. Numerous variations have been employed. Here, we use a computerized version with 4 difficulty levels (2, 3, 4, and 5 cubes) with 10 trials per level. The task consists of reproducing the pattern of cubes just presented on the computer screen by writing down the order in which the cubes appeared on a response sheet. The score is derived from the number of sequences reproduced correctly divided by the total number of sequences in the corresponding level (correct/total sequences).

##### Assessing Working Memory Updating

Transfer to working memory after training is assessed with the *n*-back task [107]. We use a computerized version of the task to assess memory updating. In this task, participants indicate whether each visual stimulus in a list matches a stimulus that occurred 1, 2, or 3 stimuli back by pressing a response key with their right hand (counterbalancing index and middle fingers to respond “yes” and “no”).

In sum, the primary outcome measures of this RCT are better performance at posttest than at pretest on a series of tasks: (1) Stroop-negative priming task, (b) oddball task, (3) *n*-back task, and (4) Corsi blocks.

### Secondary Outcomes

Improved game performance by the experimental group and the active control group between training session number 1 and 16 (final) is a secondary outcome measure. Efficacy of training will be shown by better performance in the trained video games across the training sessions. Another secondary outcome measure, maintained level of motivation and engagement, will be assessed with a questionnaire administered at training sessions 1, 8, and 16.

## Results

All analyses will be performed with the SPSS (IBM Corp) program. Statistical significance is considered as a *P* value of <.05. Analyses of variance will be conducted with 2 groups (experimental and active control) at 3 time points (pretest [baseline], posttest [postintervention], and follow-up [after 6 months without contact]). Electrophysiological data will be analyzed using EEGLAB/ERPLAB.

The preparatory phase for using the games has been completed. License to use the games has been granted by Lumosity. Participants have been recruited, pretested, and trained. The final results from this RCT are expected by the end of 2017.

## Discussion

The benefits of this RCT will be at several levels including in-depth knowledge derived from a series of carefully planned behavioral and brain imaging studies conducted to test a series of hypotheses on cognitive and brain aging. This cognitive and brain intervention study conducted in an increasingly active area of research could lead to exciting prospects in the medium and long term by exploring ways to keep older adults (mind and brain) healthy and active longer.

The findings could help prevent the negative impact of mild cognitive impairment and could have an important impact on society due to the increasing number of older adults and the associated number of neurodegenerative diseases. Cognitive decline reduces the capacity to live an independent life with associated personal, family, and social costs. Preventing the negative impact of cognitive decline could have an important effect on limited social and health care resources.

## Abbreviations

CRP: C-reactive protein
EEG: electroencephalograph
ELISA: enzyme-linked immunosorbent assay
ERP: event-related potential
ERSP: event-related spectral perturbation
ICA: Independent Component Analysis
ICT: information and communication technology
MMN: mismatch negativity
NP: negative priming
RCT: randomized controlled trial
RON: reorientation negativity
UNED: Universidad Nacional de Educacion a Distancia
